# Implementing standard antenatal care interventions: health system cost at primary health facilities in Tanzania

**DOI:** 10.1186/s12962-021-00325-0

**Published:** 2021-12-07

**Authors:** Amisa Tindamanyile Chamani, Amani Thomas Mori, Bjarne Robberstad

**Affiliations:** 1 Department of Global Public Health and Primary Care, Section for Ethics and Health Economics, University of Bergen, Bergen, Norway; 2 Department of Pharmaceutics and Pharmacy Practice, School of Pharmacy, Muhimbili University of Health and Allied Sciences, Dar es Salaam, Tanzania; 3 Chr Michelsen Institute, Bergen, Norway

**Keywords:** Cost, Antenatal care, Health system, Primary health care, Tanzania

## Abstract

**Background:**

Since 2002, Tanzania has been implementing the focused Antenatal Care (ANC) model that recommended four antenatal care visits. In 2016, the World Health Organization (WHO) reintroduced the standard ANC model with more interventions including a minimum of eight contacts. However, cost-implications of these changes to the health system are unknown, particularly in countries like Tanzania, that failed to optimally implement the simpler focused ANC model. We compared the health system cost of providing ANC under the focused and the standard models at primary health facilities in Tanzania.

**Methods:**

We used a micro-costing approach to identify and quantify resources used to implement the focused ANC model at six primary health facilities in Tanzania from July 2018 to June 2019. We also used the standard ANC implementation manual to identify and quantify additional resources required. We used basic salary and allowances to value personnel time while the Medical Store Department price catalogue and local market prices were used for other resources. Costs were collected in Tanzanian shillings and converted to 2018 US$.

**Results:**

The health system cost of providing ANC services at six facilities (2 health centres and 4 dispensaries) was US$185,282 under the focused model. We estimated that the cost would increase by about 90% at health centres and 97% at dispensaries to US$358,290 by introducing the standard model. Personnel cost accounted for more than one third of the total cost, and more than two additional nurses are required per facility for the standard model. The costs per pregnancy increased from about US$33 to US$63 at health centres and from about US$37 to US$72 at dispensaries.

**Conclusion:**

Introduction of a standard ANC model at primary health facilities in Tanzania may double resources requirement compared to current practice. Resources availability has been one of the challenges to effective implementation of the current focused ANC model. More research is required, to consider whether the additional costs are reasonable compared to the additional value for maternal and child health.

**Supplementary Information:**

The online version contains supplementary material available at 10.1186/s12962-021-00325-0.

## Introduction

In 2016, the World Health Organization (WHO) changed its Antenatal Care (ANC) guideline from a focused ANC model [1] to a standard ANC model, to reduce perinatal mortality and improve women’s experience of care. The standard model has 49 recommendations, which are grouped into five types of interventions. Compared to the focused model, the new model adds calcium supplementation throughout pregnancy to reduce the risk of pre-eclampsia (nutritional intervention), systematic screening for active tuberculosis and early ultra-sounding (maternal and fetal assessment intervention), up to 6 month dosing with sulfadoxine-pyrimethamine (SP) for preventing malaria, 7 day antibiotic regimen for asymptomatic bacteriuria (preventive measures intervention) and a minimum of eight contacts with a skilled health personnel to reduce perinatal mortality and improve women’s experience of care (health systems intervention) [2, 3]. Despite the expected benefits, there are financial concerns in developing countries, particularly in countries that did not effectively implement the simpler focused model [4–6]. Implementation of a more resource demanding standard model, need to be considered in the light of resource scarcity and other competing priorities.

The focused model recommends four visits for a normal pregnancy, i.e., one visit in each first and second trimesters and two visits in the third trimester. In this model, recommendations fall within screening, receiving therapeutic interventions and health education. The implementation manual suggests the availability of all services at the ANC unit, including rapid and easy to perform tests. It also suggests that, 30 to 40 min of personnel time should be used for the first visit, while an activity time of 20 min should be used for each of the subsequent three visits [1].Pregnant women with special needs follows an advanced version but are eligible to the basic one afterwards.

Tanzania adapted the focused ANC model in 2002, and is still using it to screen, provide therapeutic interventions and educate pregnant women [7]. Screening for malaria at the first ANC visit is an additional recommendation. The majority of pregnant women receive care from nurses and midwives at primary health facilities (dispensaries and health centers), but are referred to hospitals when advanced care is needed [7, 8]. At primary health facilities, a group health education session is normally offered in the morning, followed by individualized assessment, screening, counselling, and other interventions. During screening, some tests are conducted by nurses within the ANC unit while additional tests are performed by laboratory technicians at the facility laboratory.

Although Tanzania has documented improvement in focused ANC implementation such that 98% of pregnant women visit ANC at least once and 51% manage to complete four visits [9], there are remaining challenges. For example, only 24% of all pregnant women go for their first visit before the 4th month of pregnancy, and there are reports of underutilization, inadequate service provision, poor quality of care and scares resources [4, 7, 9–15].

Few studies have documented resources used for ANC in Tanzania and none have considered the costs of implementing the standard ANC model. Von Both (2008) estimated consultation costs from the health system perspective to be US$2.5 per visit [16], while Kowalewski (2002) estimated indirect user cost of US$9.9 per visit at primary health facilities [17]. A study by Saronga (2014) in rural areas of southern Tanzania, reported a cost of US$16.4 per visit from a health system perspective [18]. In 2015, a study in the neighboring country Rwanda estimated a cost of US$10.65 per visit [19] under the focused model and US$9.9 per visit after addition of some recommendations for the standard model [20], both from a health system perspective.

While the WHO's recommendations influence guideline updates in developing countries, country-level economic evidence to support such decisions are scare. Therefore, our aim was to estimate the cost of providing ANC services at primary health facilities in Tanzania under two scenarios: (1) the current practice, which reflects a suboptimal implementation of the focused ANC model, and (2) a hypothetical full implementation of new recommendations from the standard ANC model.

## Methodology

### Study settings

We conducted this study in Kigamboni and Korogwe districts. Kigamboni is situated along the Indian Ocean, south-east of Dar es Salaam (the largest city) and is divided into nine wards with a population of 206,000 [21]. Kigamboni has a total of 21 public health facilities, including one designated hospital, two health centres and 18 dispensaries. Korogwe district is within Tanga region, 283 km north of Dar es Salaam and has a population of about 260,000. Korogwe district provides health services from one district hospital, three public health centers and 44 dispensaries.

From each district, we purposively chose three primary health facilities i.e., one health centre and two dispensaries. We selected facilities which attended many pregnant women for a year, to estimate maximum resources used for the focused model, and maximum resources required for the standard model. Both dispensaries and health centres provide all basic ANC services in Tanzania. A dispensary is the lowest post and offers mainly outpatient services, while a health center provides more services and to a larger population.

### Data collection

We collected data from a health system perspective using a micro-costing or bottom-up approach. This perspective excludes patients costs of accessing health care such as transportation, cost-sharing, or time off work. Data collection involved documentation review, physical inventory, and interviews with healthcare workers. We identified actual resources used to provide focused ANC for one financial year, from July 2018 to June 2019. For the standard ANC model, we used an implementation manual to model additional resource that would be required, since the intervention is not yet implemented in Tanzania and observational data is consequently unavailable. We classified resources consumed within 1 year as recurrent cost; these included personnel, non-medical and medical supplies, medicines, laboratory supplies, utility, maintenance, and repair of capital items. We categorized resources that last more than a year as capital cost items, which included buildings, equipment, furniture and national level programme cost [22].

### Quantification and valuation of resources

#### Personnel

To cost the focused ANC, we interviewed each involved health personnel identified, about his/her qualifications, salary scale, allowances, and a proportion of their working time devoted to ANC compared to other duties. We assumed that pregnancies are evenly distributed over the year and that time allocated to ANC did not vary over the study period. We included gross salary, overtime and uniform allowances when calculating personnel costs. We excluded annual leave cost because data were unreliable. We used gross salary rates for a medical attendant to value the effort of skilled and trained volunteers working as medical attendants. For each personnel, we multiplied the proportion of time allocated to ANC with the annual cost of a full position to estimate personnel cost attributed to ANC. For non-medical personnel (guards and cleaners), we used the proportional floor space for ANC activities as allocation factor.

The WHO did not recommend activity time for the standard ANC model as they did for the focused model. Likewise, there is no recommended time for administrative duties related to ANC under the focused model. We assumed that the time allocated to ANC by nurses and laboratory personnel was the total of activity time and the time for administrative duties. We calculated a ratio between the allocated time (calculated above) and the activity time (suggested by the WHO) of 40 min (first visit) and 20 min (subsequent visits). This ratio indicated the proportion of allocated time that was used for administrative duties, assuming the activity time used resembled the suggestion by the WHO. To calculate personnel time for the standard model, we first assumed that the activity time use per visit for focused ANC would apply also to the standard model. We then used the ratio (calculated above) to factor in the time that will be used for administrative duties. With these inputs, we could derive additional personnel time required to switch from focused to standard ANC for each of the included facilities and per pregnancy. We used the annual cost for nurses at each facility to value the additional time use of introducing standard ANC. For other personnel we assumed that time allocated to ANC will not change with a new model. To account for a new routine ultrasound scan we included the annual cost of a sonographer at each facility and assumed 50% allocation to ANC.

#### Supplies

We used ANC monthly reports to quantify medicine and laboratory supplies consumed. Monthly reports record types and numbers of tests performed, vaccines provided, and medicines administered. For example, with a report of 260 doses of Tetanus Toxoid vaccines, we estimated 13 vials of the vaccine (one vial contains 10 mls and the standard dose is 0.5mls) and 260 syringes of 1 ml each. We used the standard ANC recommendations, shown in Table 1**,** to quantify additional supplies, including calcium supplements for 6 months and seven days antibiotic for asymptomatic bacteriuria. We assumed that 13% of pregnant women have asymptomatic bacteriuria in Tanzania [23] and therefore would receive treatment. We quantified other medical supplies (gloves, cotton wool, spirit, bedsheet, and safety boxes) issued to ANC and the laboratory from pharmacy registers, and additional supplies for a standard model based on the recommendations**.** The allocation of shared medical supplies reflected nurses and laboratory staff time allocation to ANC. We also included thermal and tissue papers and ultrasound jelly for a routine scan.

**Table 1:** A comparison of recommendations between the focused and the standard antenatal care models

Output per pregnancy	Focused ANC (Tanzania)	Standard ANC
Number of visits	4	8
Anemia test by hemoglobinometer	4	3
Urine dipstick test	4	3
Blood grouping test	1	1
Malaria by rapid diagnostic test (mRDT)	1	1
Syphilis test	2	2
HIV test	2	2
Tetanus Toxoid vaccine	Depend on the previous vaccination status	
Seven-day antibiotic for asymptomatic bacteriuria	0	13% of the population
Monthly Sulfadoxine Pyrimethamine for malaria	≥ 3	6
Long-Lasting Insecticide Treated Net (LLITN)	1	1
Ferrous and folic acid supplements(months)	≥ 3	6
Antihelminthes (Albendazole 400 mg)	1	1
Calcium supplementation (months)	0	6
Routine ultrasound scan	0	1

We interviewed either a facility accountant or facility in-charge on expenses incurred on cleaning detergents and equipment (bucket, broom, moppers) and allocated them using the same factor as for cleaners (see above). We quantified stationaries used at the laboratory and ANC, and we assumed that consumption would double under the standard ANC model. We increased the total quantity for each recurrent supply by 5% for spoilage, which is common in applied costing studies [24]. We used the Medical Store Department (MSD) price catalogue (2018/2019) and local market prices to identify values. These costs were further adjusted upward by 10% to account for local transport from the supplier [24]. The annual cost was estimated as the product of adjusted quantities, adjusted unit cost and allocation to ANC for each supply.

#### Water, gas, and electricity

We interviewed the facility accountant or facility-in charge about water, gas, and electricity bills. We allocated 50% of the gas cost (used to store vaccines during electricity cuts of) to ANC because the freezer also stores vaccines used for other programmes. We used the proportion of equipment and rooms for ANC unit to allocate electricity and water bills for the focused model. For the standard model, we also adopted the electricity bill from a facility installed with an air condition at ANC unit to other facilities, to cater for air conditioning the ultrasound room.

#### Capital items

We used the MSD price catalogue or local market prices to assign values to identified equipment and furniture. The proportion of time allocated to ANC by personnel involved with specific equipment and furniture was used as the allocation key. For the standard ANC model, we in addition added annual costs for the ultrasound machine, examination bed, movable chair, washbasin stand, double step, and air-condition. These were allocated to ANC by 50%, an assumption made in the WHO implementation manual for standard ANC [2]. Assumptions about useful life years for capital items were adopted from the WHOs “Choosing Interventions that are Cost-Effective project” [25], and in addition we assumed 10 years for the ultrasound machine. We calculated annual costs using a 5% interest rate [22].

We used a tape measure to estimate total floor space of each facility and subsequently allocated it to ANC using personnel time. We adapted the costs used to construct a Reproductive and Child Health (RCH) building in Korogwe in 2008, assuming 20 years of useful life and 5% interest rate. Observations made during data collection support the idea that the standard model can be facilitated within buildings already available, with minor renovations for the ultrasound machine and its security. Finally, we added 5% of the total capital cost for maintenance and repair and then 10% of the total annual cost at facility level represented national level programme cost [26].

### Data analysis

We adapted the data collection tools from the Costing Guidelines for HIV Prevention Strategies [27], and used Microsoft Excel® for compilation and analysis. We calculated the total cost by aggregating annual cost of all supplies, personnel, and capital goods. We calculated the unit cost per visit as the ratio of total costs to the number of ANC visits, and the cost per pregnancy as the ratio of total cost to the number of first visits. Items were valued in TSh and converted to US$ using the Bank of Tanzania exchange rate for early July 2018 (US$1 = TSh2278) [28].

## Results

### ANC characteristics of study facilities

We recorded a total of 19,342 visits across six primary health facilities, it is 90% (19,342/21,080) of total visits expected with full compliance of the focused model (Table 2). Pregnant women at a health centre in Kigamboni (health centre_1_) attended more than the recommended four visits. Possibly some women were referred from dispensaries for specialized care including ultrasound.

**Table 2:** Number of Antenatal care visits recorded  and estimated at each study facility

	Focused ANC^a^			Standard ANC^b^
Health facility	First visits	Annual visits	Visit/ pregnancy	Annual visits
Health centres				
Health centre_1_	1,676	7714	4.6	13,408
Health centre_2_	742	2149	2.9	5936
Dispensaries				
Dispensary_1_	795	2735	3.4	6360
Dispensary_2_	1019	3886	3.8	8152
Dispensary_3_	397	1140	3	3176
Dispensary_4_	641	1718	2.7	5128
All facilities	5270	19,342		42,160

^a^Observed actual numbers

^b^Estimated assuming 8 visits per pregnancy

### Personnel time

Table 3 presents the observed time (in hours) allocated to focused ANC by nurses and laboratory staff at the six health facilities under the focused model and additional time required for the standard model. The ratio between allocated time and activity-time for the focused model suggests that time use for personnel was 1.6 to 3.8 times higher compared to what was suggested by the WHO. At health centre_1_ with a ratio of 1.66, the intervals between one attendance and the other were 66 min for a first visit and 33 min for a revisit. While at dispensary_1_ with a ratio of 3.75, the intervals were 2 h and 30 min for a first visit and 75 min for a revisit. Within current levels of personnel productivity, facilities need between 1,145 and 4535 h of personnel per year to implement standard ANC model, equivalent to about one and three additional nurses. Each pregnant woman will require between 1.8 and 5.7 additional hours of personnel time throughout pregnancy.

**Table 3:** Time (hours) used for the focused model and time required for the standard model

	Health center (2)		Dispensaries (4)			
	HC_1_	HC_2_	Dispensary_1_	Dispensary_2_	Dispensary_3_	Dispensary_4_
A. Allocated personnel time for focused ANC	5184	2112	4416	3744	864	1248
B. Recommended Activity-time_1_-focused ANC	3130	964	1177	1635	512	786
C. Ratio (A/B)	1.66	2.19	3.75	2.29	1.69	1.59
D. Activity time _2_—standard-ANC	5028	2226	2385	3057	1191	1923
E. Personnel time for standard ANC (C*D)	8328	4879	8951	7000	2009	3052
F. Additional time (E-A)	3144	2767	4535	3256	1145	1804
G. Additional time per pregnancy	1.88	3.73	5.70	3.20	2.88	2.81

### Annual cost and unit cost

The detailed costing for recurrent and capital items under the focused and the standard models are shown in the Additional file 1. Health facilities in Kigamboni (Health centre_1_, dispensary_1and 2_) recorded more visits and higher cost compared to health facilities in Korogwe (Health centre_2_ Dispensaries_3 and 4_). The results in Table 4 suggest that the cost will increase from US$30,880 to US$59,715 with implementation of a standard model, at each primary health care facility recording more than 1000 ANC visit per year. More specifically, the cost will increase from US$40,172 to US$75,898 at health centres and from US$26,234 to US$51,263 at dispensaries. The unit cost per pregnancy will increase from about US$33 to about US$63 at health centres, and from about US$37 to US$72 at dispensaries. The unit cost per visit will not change substantially with guideline update, the increased costs are largely attributable to increased number of visits.

**Table 4:** A comparison of antenatal care cost between  focused and standard antenatal care models at health centres and dispensaries in Tanzania

Cost in US$	Health centres (2)		Dispensaries (4)		All facilities (6)	
	Focused ANC	Standard ANC	Focused ANC	Standard ANC	Focused ANC	Standard ANC
Personnel	30,483 (38%)	50,591 (33%)	38,116 (36%)	73,186 (35%)	68,599(37%)	123,777(35%)
Medicine/medical supplies	21,846 (27%)	49,206 (32%)	25,441 (24%)	59,746 (29%)	47,287(26%)	108,952(30%)
Laboratory supplies	11,076 (14%)	24,649 (16%)	15,744 (15%)	30,028 (15%)	26,821(14%)	54,676(15%)
Other recurrent items	2,146 (3%)	4,449 (3%)	3,978 (4%)	9,172 (4%)	6,124(3%)	13,621(4%)
Capital items	14,793 (18%)	22,901 (15%)	21,658 (21%)	34,363 (17%)	36,451(20%)	57,264(16%)
Total cost	80,344	151,797	104,938	206,493	185,282	358,290
Cost per facility	40,172	75,898	26,234	51,623	30,880	59,715
Cost per ANC visit	8.1	7.8	11.1	9.1	9.6	8.5
Cost per pregnancy	33.2	62.8	36.8	72.4	35.2	68

The costs for all cost categories increased relatively proportional when moving from a focused to a standard ANC model. For both models, personnel cost was the most important accounting for a third and above of the total cost, followed by medicine and medical supplies, capital items, laboratory supplies and other recurrent items. Additional personnel time and the cost of a sonographer increased annual personnel cost. Calcium supplementation for 6 months (US$22,445 across facilities), up to six doses of SP, seven-day antibiotics to 13% of the population and other medical supplies, doubled medicine and medical supplies cost.

## Discussion

Our findings show that, more resources are required to implement the standard ANC model at primary health facilities in Tanzania. At each health centre with more than 2000 ANC visits annually, two additional nurses will be required, while one to three additional nurses will be required at dispensaries with more than 1000 annual visits. Also, the resources required for medical and laboratory supplies would need to double. It is important to emphasize that results for the standard ANC model were extrapolations built on facility data.

The difference between unit cost per visit at a health centre (US$8.1) and a dispensary (US$11.1) was small in our study. This contrasts with estimates from Mtwara in South of Tanzania, where unit costs were substantially higher at health centres (US$19) than at dispensaries (US$ 4.75) [18]. On the other side, our estimates are relatively similar to a study from Rwanda, where unit costs for the focused model were estimated to be US$ 10.65 and US$10 for the standard model [19, 20].

Personnel time was the main cost driver for both ANC models, and represented more than one-third of the total cost at the health centre and dispensary. Our result shows allocated time exceeded activity time differently at each facility, including time for administrative duties. A simulation study in Tanzania estimated the activity time of 46 min and 36 min for counselling and few test at ANC for the first visit and subsequent visits respectively [29], while observational studies have reported the activity time of less than 20 min for each visit [30]. Our analysis was based on self-reporting, and it was not possible to differentiate the activity time and the time for administrative duties, which would have required a time sequence study. But we see the potential to improve personnel productivity at some facilities, which could reduce personnel cost for the standard model if appropriately addressed. This argument is based on our data that shows large variance across facilities in the ratio between allocated time and activity time across facilities.

Resources required for medicine, medical and laboratory supplies will be more than two times for both health centres and dispensaries with implementation of standard ANC. Calcium supplementation is one of the drivers, despite that it was not cost effective in Ethiopia [31]. Our data indicate that, coverage for medicines and laboratory tests was still not optimal seventeen years after adoption of the focused model. Coverage was lower at facilities in Korogwe (health centre _2_ Dispensary _3 and 4_) compared to facilities in Kigamboni (health centre _1_ Dispensary _1 and 2_) (Additional File 1). The stock status has been one of the challenges to effective coverage of the focused ANC recommendation. Therefore, economic evaluation and implementation studies of new strategies to ensure availability of supplies are important before adoption of a more resourceful model.

Our estimation of unit costs for the standard model assumes that number of pregnancies will not change. Our costing included resources required to provide ultrasound under the standard model. It is not clear if ultrasound use will improve fetal and maternal outcomes [32], but it is possible that standard ANC will improve adherence, which subsequently could improve health outcomes and reduce unit costs per visit. This study was undertaken in facilities which attended many pregnant women annually. For smaller health facilities, the unit cost of ANC per pregnancy could be somewhat higher.

## Study limitations

This study has several limitations, first, we purposively sampled six relatively large primary health facilities attending many pregnant women. The plan was to estimate the highest possible annual cost, which can be used by policy makers when budgeting for the standard ANC model. However, the approach also limits the generalizability of our findings in Tanzania, especially to facilities attending few pregnant women. We also estimated resources for an ambition to fully implement the standard ANC model. Second, incomplete, or inaccurate facility records compelled us to exclude some information, such as the cost of managing sick pregnant women, which represents potential underreporting. Third, we used self-reporting to allocate personnel time to antenatal care which is prone to information bias. This could have resulted into overestimation or underestimation of personnel cost. Nevertheless, personnel were asked to allocate time to each of their responsibilities, which might have reduced the bias. Finally, we considered cost from a provider’s perspective, while ignoring the additional cost imposed on pregnant women from more facility visits. This may underestimate the overall societal cost implication of policy change from focused to standard antenatal care.

## Conclusion

The introduction of standard ANC in primary health facilities in Tanzania may double the resources requirements compared to current practice under the assumption that all resources are being efficiently used. While resource availability has been a challenge for effective implementation of the focused ANC model, more research is required, to consider whether the cost of implementing standard ANC model are reasonable compared to the additional value for maternal and child health.

## Supplementary Information

**Additional file 1.** Annual costs for recurrent and capital items under the focused and the standard ANC models.

## Data Availability

All data generated or analysed during this study are included in this published article.

## Abbreviations

ANC: Antenatal care
WHO: World Health Organization
