# Supplementary material for: Implementing standard antenatal care interventions: health system cost at primary health facilities in Tanzania
Source: Cost Eff Resour Alloc. 2021 Dec 7;19:79. doi: 10.1186/s12962-021-00325-0 (PMC8650535; doi:10.1186/s12962-021-00325-0)
Supplement: Supplementary file 1 — Additional file 1. Annual costs for recurrent and capital items under the focused and the standard ANC models. [file 12962_2021_325_MOESM1_ESM.docx]

**Additional file 1**: **Annual costs for recurrent and capital items under the focused and the standard ANC models.**

|  | Annual cost (US$) in Health Centre (n=2) | | | | | | Annual cost (US$) in Dispensaries (n=4) | | | | | | | | | | Annual cost (US$) all facilities (n=6) | |
| --- | --- | --- | --- | --- | --- | --- | --- | --- | --- | --- | --- | --- | --- | --- | --- | --- | --- | --- |
|  | Health Centre_1_ | | Health Centre_2_ | | Subtotal for health centers | | Dispensary._1_ | | Dispensary._2_ | | Dispensary._3_ | | Dispensary_4_ | | Subtotal for dispensaries | |  |  |
|  | FANC | S ANC | F ANC | S ANC | F ANC | S ANC | F ANC | S ANC | F ANC | S ANC | F ANC | S ANC | F ANC | S ANC | F ANC | S ANC | F ANC | S ANC |
| Recurrent item |  | | | | | | | | | | | | | | | | | |
| Personnel | 17,524 | 28,052 | 12,959 | 22,540 | 30,483 | 50,591 | 16,494 | 30,324 | 13,635 | 24,148 | 2,513 | 6,253 | 5,474 | 12,460 | 38,116 | 73,186 | 68,599 | 123,777 |
| Medicine and medical supplies | 17,817 | 33,769 | 4,029 | 15,437 | 21,846 | 49,206 | 7,873 | 18,649 | 10,067 | 20,985 | 1,775 | 7,459 | 5,726 | 12,653 | 25,441 | 59,746 | 47,287 | 108,952 |
| Laboratory supplies | 8,459 | 15,285 | 2,617 | 9,363 | 11,076 | 24,649 | 3,747 | 7,087 | 5,801 | 10,237 | 603 | 3,417 | 5,593 | 9,286 | 15,744 | 30,028 | 26,821 | 54,676 |
| Non-medical supplies | 185 | 408 | 68 | 153 | 253 | 560 | 376 | 769 | 316 | 656 | 52 | 113 | 71 | 157 | 815 | 1,695 | 1,067 | 2,255 |
| Utility | 1,229 | 2,458 | 290 | 975 | 1,519 | 3,433 | 1,159 | 2,713 | 1,208 | 2,810 | 144 | 682 | 47 | 490 | 2,558 | 6,695 | 4,076 | 10,128 |
| Repair and maintenance | 195 | 235 | 179 | 221 | 374 | 456 | 196 | 238 | 166 | 208 | 115 | 164 | 129 | 171 | 606 | 782 | 980 | 1,238 |
| **Subtotal** | **45,410** | **80,207** | **20,141** | **48,688** | **65,551** | **128,896** | **29,845** | **59,782** | **31,193** | **59,045** | **5,202** | **18,089** | **17,040** | **35,216** | **83,280** | **172,131** | **148,831** | **301,027** |
| Capital items |  | | | | | | | | | | | | | | | | | |
| Building | 1,245 | 1,245 | 1,419 | 1,419 | 2,664 | 2,664 | 1,849 | 1,849 | 1,701 | 1,701 | 1,211 | 1,211 | 1,211 | 1,211 | 5,972 | 5,972 | 8,636 | 8,636 |
| Equipment and furniture | 2,663 | 3,457 | 2,162 | 3,002 | 4,825 | 6,459 | 2,079 | 2,919 | 1,624 | 2,464 | 1,083 | 2,075 | 1,360 | 2,201 | 6,146 | 9,659 | 10,971 | 16,118 |
| Programme cost | 4,932 | 8,467 | 2,372 | 5,311 | 7,304 | 13,778 | 3,377 | 6,431 | 3,452 | 6,300 | 750 | 2,137 | 1,961 | 3,863 | 9,540 | 18,732 | 16,844 | 32,510 |
| **Subtotal** | **8,839** | **13,169** | **5,953** | **9,732** | **14,793** | **22,901** | **7,305** | **11,199** | **6,777** | **10,465** | **3,044** | **5,423** | **4,532** | **7,275** | **21,658** | **34,363** | **36,451** | **57,264** |
| **Total cost** | **54,250** | **93,377** | **26,094** | **58,420** | **80,344** | **151,797** | **37,150** | **70,981** | **37,970** | **69,510** | **8,246** | **23,512** | **21,572** | **42,491** | **104,938** | **206,493** | **185,282** | **358,290** |

**Key** FANC-Focused Antenatal Care

SANC-Standard Antenatal Care
